# Effects of maximal strength training on bone mineral density in people living with HIV and receiving anti-retroviral therapy: a pilot study

**DOI:** 10.1186/s13102-020-00216-6

**Published:** 2020-10-23

**Authors:** Enock M. Chisati, Demitri Constantinou, Fanuel Lampiao

**Affiliations:** 1 Department of Physiotherapy, College of Medicine, University of Malawi, Blantyre, Malawi; 2 Consortium for Advanced Research Training in Africa, Nairobi, Kenya; 3 Center for Exercise Science and Sports Medicine, FIMS Collaborating Center of Sports Medicine, University of the Witwatersrand, Johannesburg, South Africa; 4 Department of Biomedical Sciences, College of Medicine, University of Malawi, Blantyre, Malawi; 5 Africa Centre of Excellence in Public Health and Herbal Medicine, Blantyre, Malawi

**Keywords:** Bone mineral density (BMD), Maximal strength training (MST), People living with HIV (PLWHIV), Anti-retroviral therapy (ART), Exercise

## Abstract

**Background:**

Anti-retroviral therapy (ART) is associated with low bone mineral density (BMD) among people living with HIV (PLWHIV). Although physical activity is recommended for improving bone health in patients with reduced BMD, data on effects of strength exercises on low BMD among PLWHIV is scarce. This study therefore aimed to determine the effects of a 12 weeks maximal strength training (MST) on BMD among PLWHIV in Blantyre, Malawi.

**Methods:**

Twenty-six PLWHIV with reduced BMD were randomised into a training group (TG, *n* = 15) and control group (CG, *n* = 11). The TG underwent 12 weeks of MST consisting of 4 sets of 3 to 5 repetitions at 85–90% of one repetition maximum (1RM) 3 times per week. The CG was advised to maintain their usual lifestyle. Measurements of BMD using dual-energy X-ray absorptiometry, 1RM using a squat machine, heart rate using a heart rate monitor, weight, height and body mass index were obtained before and after the intervention in the TG and CG. Descriptive statistics and student’s t - tests were used to analyse data.

**Results:**

The study was conducted for 12 weeks. Data of 24 participants [14 (TG) and 10 (CG)] were analysed. At base line, there were no significant differences in age (*p* = 0.34), height (*p* = 0.91), weight (*p* = 0.43) and body mass index (*p* = 0.34) between participants in the TG and the CG. After the intervention, there were significant improvements in lumbar BMD (*p* < 0.001) and resting heart rate (*p* = 0.03) in the TG compared to the CG. There were significant improvements in muscle strength (1 RM) in both the TG (*p* < 0.001) and the CG (*p* = 0.01).

**Conclusions:**

MST improves lumbar BMD and strength in PLWHIV receiving ART in Blantyre, Malawi. MST with a shorter exercise duration of 12 weeks seem to have the potential in treating reduced BMD in PLWHIV.

**Trial registration:**

PACTR201712002889203. Registered with the Pan African Clinical Trial Registry on 22nd December, 2017 at www. pactr.org

## Background

The use of antiretroviral therapy (ART) to treat human immunodeficiency virus (HIV) leads to reduced bone mineral density (BMD) [1–3]. Low BMD categorised as osteopaenia and osteoporosis may increase the incidence of fractures among people living with HIV (PLWHIV) [4] which may escalate the risk for morbidity and increase mortality. In addition to providing calcium supplements and vitamin D, pharmacological approaches through the provision of Teriparatide, Denosumab and Bisphosphate drugs have been proposed as methods of managing bone demineralisation occurring due to ART [5]. Although promoted in the management of reduced BMD, side effects associated with pharmacological approaches and calcium supplements as well as adherence problems may limit the use of such strategies among PLWHIV [6].

Among other recommendations, physical activity and exercises are included in guidelines for preventing bone loss even among PLWHIV [7–9]. Evidence has shown the beneficial effects of some physical activities such as dancing, walking, weight lifting and jogging in preventing and managing bone demineralisation [10]. However, evidence that high bone mineral density is linked to physical activity is mostly misinterpreted as evidence that any physical activity will lead to increased BMD [11]. Despite some evidence that increases in BMD could result from any physical activity [12], high force weight bearing physical activities lead to pronounced increases in bone mineral density [10]. Therefore, the intensity and type of the physical activity has an important impact on BMD.

Increases in BMD following strength exercises has been reported in a number of studies [7–9, 13]. Thus strength exercise may be used as a strategy to increase BMD in PLWHIV and receiving ART [7]. Compared to pharmacological approaches, exercise interventions are associated with higher adherence rates in managing bone demineralisation [14]. Specifically, strength exercises have shown a higher compliance rate [15] with an adherence rate of over 80% in randomised controlled trials [16]. Reports also reveal an increased adherence among participants performing facility based exercises with shorter durations [14] such as maximal strength exercises [17, 18]. Thus shorter and facility based strength exercise programmes targeting BMD may be appropriate in increasing adherence.

Despite evidence that strength exercises are effective in increasing BMD in men and women [18–21], knowledge on the effects of exercise on low BMD among PLWHIV and receiving ART is scarce. Further, strength exercises have been proven to be safe, practical, inexpensive and beneficial in improving metabolic outcomes among PLWHIV [22–25]. In view of declining rates of mortality and morbidity among PLWHIV due to increased accessibility of ART in resource limited settings, exercise may be a cost effective non pharmacological strategy in preventing bone loss thereby reducing osteoporosis and fall related fractures. However, despite reports of increases in bone loss due to ART [26, 27], there is still lack of information on the optimal mode of frequency, duration and intensity of strength exercise on BMD in PLWHIV [22, 28]. The aim of this study therefore was to determine the effects of a 12 weeks maximal strength training (MST) exercise programme on BMD among PLWHIV in Blantyre, Malawi.

## Methods

### Study design and setting

This was a parallel randomised controlled pilot study conducted at the College of Medicine Sports Complex in Blantyre city, Malawi. The Sports Complex houses a gym quipped with a variety of exercise training equipment and accommodates about 20 participants per day. It operates daily from 8.00 am to 10.00 pm. Different people within Blantyre city patronize the facility to engage in different exercises either for health or sports performance.

### Study population

Participants were recruited from Queen Elizabeth Central Hospital (QECH) in Blantyre Malawi. Male and female adults aged 18–45 years living with HIV and receiving ART who had reduced BMD were included in the study. The World Health Organisation (WHO) recommends the use of Z-scores (defined as an individuals’ BMD in comparison to age-matched normal individuals) in reporting BMD for premenopausal women or men less than 50 years of age and children [29]. A Z-score of − 2.0 or lower is defined as low BMD for chronological age or below the expected range for age whereas a Z-score above − 2.0 is within the expected range for age [30–32].

Participants were included if they were receiving tenofovir based ART regimens for more than 12 months and had reduced BMD. The more than 12 months duration was chosen because reductions in BMD are more pronounced after this period [33]. Participants with contraindications to exercise (such as serious cardiorespiratory, neurological or orthopaedic conditions which would limit participation to the exercise regimen), and were taking any calcium supplements and pharmacological therapies were excluded.

### Exercise protocol

Participants were randomly allocated to either an exercise training group (TG) or control group (CG). Participants in the TG followed a MST programme for 12 weeks comprising three sessions each week with a total of 36 sessions under the supervision of a qualified physiotherapist. The participants were instructed not to add any leisure exercises that included high impact jumping and lifting heavy loads during the study period. The MST sessions consisted of squat exercises performed on a hack squat machine (Model HLS2000) using the lower extremities. Before the main exercise session, participants performed a warm-up comprising two sets of 8 to 12 repetitions at approximately 50% of the participant’s training load. The warm up was followed by the main exercise consisting of four sets of 3 to 5 repetitions at 85 to 90% of one repetition maximum (1RM). A break of 2 to 3 min was allowed between the sets. Execution of the exercise started from a straight legs position, down to a 90^o^ angle in the knee joint and up again. Participants’ 1RM was re-evaluated every week to guide progression of the intensity of the exercise. Participants in the CG were instructed to keep living their usual lifestyle during the study period.

For effective supervision, each participant was scheduled his or her own time for the exercise. The physiotherapist who was supervising the exercise regimen was blinded from knowing that the participants were in a study and the purpose of the study to obtain reliable results.

### Randomisation

To allocate participants to either TG or CG, a random sequence of numbers was generated from the computer using the RANDBETWEEN function in Microsoft Excel, 2016. Treatments were then allocated to participants in sequence using numbered opaque envelopes containing the treatment allocations. The generation of the number sequence and allocation of the envelops to the participants was done by a Physiotherapist who was not involved in data collection and evaluation of the outcomes.

### Data collection and equipment

Data was collected from June, 2018 to March, 2019. Variables of BMD (*g*. *cm*^−2^), maximal strength, heart rate, weight, height and body mass index (BMI) were obtained and recorded on a data collection form before and after the exercise programme in the TG and the CG to determine effects of the exercise.

#### Bone mineral density

Femoral neck and lumbar spine BMD (*g*. *cm*^−2^) was measured using dual-energy X-ray absorptiometry (DEXA) (Hologic Discovery-Wi (S/N 84668), software version 13.5.3.2:5, Hologic Bedford Inc., Bedford, MA, USA) at QECH. Femoral neck BMD was measured at the left hip only. Measurement of the lumbar spine BMD was done from the first to fourth lumbar spines and a total BMD value was recorded.

#### Maximal strength

Maximal strength, obtained as 1RM was measured on the squat machine as described under the exercise protocol section. To determine 1 RM, several lifts were executed with an increasing load of 5 kg for each lift until the highest load lifted was reached.

#### Body weight, height and heart rate

Body weight (kg) and height (cm) measurements were obtained using a Stadiometer (HS – he DBS00361, Model: 1127154) following the manufacturer’s guidelines. A heart rate monitor (Polar FT4, Model C317T21559445) was used to obtain heart rate measurements before and after the exercise.

#### Body mass index

BMI for each participant was calculated by dividing weight measurement by the square of the height measurement in meters (m^2^).

### Sample size

Sample size estimates are based on the effect size, alpha and number of participants in each group. From the results, an increase of 3% in femoral neck BMD and 4% in lumbar BMD after exercise training was obtained. To detect a 3% increase in mean BMD, with an alpha of 0.05 at 80% power using a two - sided test, 15 participants in each group were required. Sample size calculations were done using a G*power 3.1.6 computer software programme.

### Data analysis

Statistical Package for the Social Science (SPSS version 25) was used to analyze the data. Descriptive statistics such as mean and standard deviation (SD) were used to characterize the data variables. All data variables were normally distributed. Student’s t - test was used to analyse differences between and within the groups. All statistical tests were two - sided and a *p* value of ≤0.05 was considered statistically significant.

### Ethical considerations

As participants came for refilling their ART medication at QECH, they were requested to attend a health talk regarding the purpose of the study and invited to participate. This health talk was conducted by the researchers. Willing participants were directed into a separate room where the aim and objectives of the study were again explained and screening for eligibility was done. Written informed consent was obtained from eligible and willing participants.

All ethical procedures were followed and privacy and confidentiality were ensured by allocating codes to the participants. The study was approved by the University of Malawi’s College of Medicine Research and Ethics Committee (COMREC) registration number P.06/17/2206. The study was registered with the Pan African Clinical Trial Registry on 22nd December, 2017 with identification number: PACTR201712002889203.

## Results

**Fig. 1:**
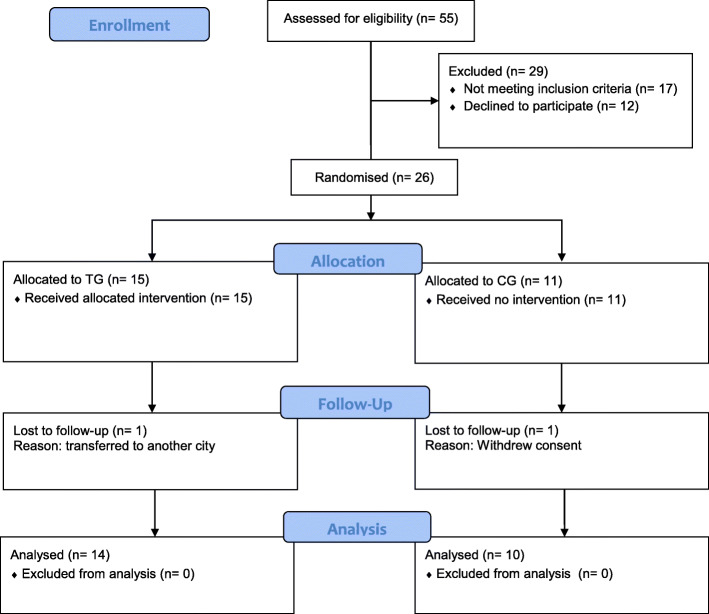
Study flow diagram showing numbers of participants in the TG and CG

Out of 55 eligible participants, 29 participants were excluded because they either did not meet the inclusion criteria or they declined to participate. Twenty-six participants were therefore included and randomly allocated to either a TG (*n* = 15) or CG (*n* = 11). Two participants (one from each group) did not finish the training due to transfer and withdrawal of consent (Fig. 1). The study adhered to CONSORT guidelines for conducting randomised controlled trials. The training group completed all the planned training sessions. No significant differences in baseline characteristics were observed between participants in the TG and the CG (Table 1).

**Table 1:** Characteristics of participants at baseline

	TG (*n* = 14)	CG (*n* = 10)	*P* – value
Age (years)	35.1 ± 6.7	37.7 ± 6.3	0.34
Height (cm)	160.1 ± 0.1	159 .7 ± 0.1	0.91
Weight (kg)	57.0 ± 7.8	60.1 ± 11.3	0.43
BMI (kg/cm^2^)	22.2 ± 2.5	23.7 ± 4.7	0.34

Data are presented as mean ± SD, *TG* Training group, *CG* Control group, *P*-values are based on Independent samples t – test

On average, there were significant improvements in lumbar spine BMD and 1RM (*p* < 0.001) as well as in resting heart rate (*p* = 0.027) in the TG participants after 12 weeks (paired samples t-test). Only 1RM was significantly higher (*p* = 0.013) after 12 weeks in the CG (Table 2).

**Table 2:** Changes in physiological parameters before and after exercise training

	TG (*n* = 14)		CG (*n* = 10)	
	Pre-training	Post-training	Pre-training	Post-training
Femoral neck BMD (*g*. *cm*^−2^)	0.770 ± 0.1	0.778 ± 0.1	0.772 ± 0.1	0.786 ± 0.1
Lumbar spine BMD (*g*. *cm*^−2^)	0.771 ± 0.1	0.804 ± 0.1*	0.774 ± 0.7	0.780 ± 0.1
1 RM (kg)	106.79 ± 31.4	222.14 ± 43.2*	110.70 ± 34.8	123.00 ± 33.2
Heart rate (bpm)	87.00 ± 16.4	78.86 ± 11.2*	74.30 ± 13.7	76.20 ± 10.3

Data are presented as mean ± SD, *TG* Training group, *CG* Control group, *bpm* beats per minute, * Significant difference *p* < 0.05

The changes in mean values for lumbar spine BMD (0.006 *g*. *cm*^−2^ vs 0.033 *g*. *cm*^−2^) and heart rate (1.9 bpm vs − 8.14 bpm) were significantly higher within the TG compared to the CG after 12 weeks. Whereas the mean value for 1RM (12.3 kg vs 115.36 kg) was significantly higher in both the TG and CG after 12 weeks (Table 3).

**Table 3:** Mean differences in physiological parameters between and within the groups

	Within groups*		Between groups#
	TG (*n* = 14)	CG (*n* = 10)	
Femoral neck BMD (*g*. *cm*^−2^)	0.008 (0.472)	0.014 (0.144)	0.006 (0.675)
Lumbar spine BMD (*g*. *cm*^−2^)	0.033 (< 0.001)	0.006 (0.568)	0.027 (0.026)
1 RM (kg)	115.36 (< 0.001)	12.30 (0.013)	103.06 (< 0.001)
Heart rate (bpm)	–8 .14 (0.027)	1.90 (0.561)	−10.04 (0.044)

Data are presented as mean difference (*p*-value), *TG* Training group, *CG* Control group, *bpm* beats per minute, *P*-values are based on *Paired samples t-test, #Independent samples t-test

## Discussion

The main purpose of this pilot study was to determine the effects of 12 weeks of MST on reduced BMD in HIV infected individuals receiving ART in Blantyre, Malawi. Results reveal that MST performed three times a week for 12 weeks is effective in increasing lumbar spine BMD among PLWHIV on ART. The MST intervention also caused improvements in muscle strength among the participants.

Current findings are in line with the study by Santos et al. (2015), who demonstrated that a 12 weeks progressive exercise programme was appropriate to impact significant lumbar and femoral neck bone increases in 20 individuals living with HIV^9^. Despite some evidence that all physical activity could be important in increasing BMD [12], weight bearing physical activities with high loads, yield a notable increase in BMD [10]. Although bones respond to small loading stimuli, evidence indicate that high mechanical loading, such as those used in MST, increase BMD by acting on muscle and ground reaction forces which in turn induces mesenchymal stem cells osteogenic differentiation towards osteoblasts [13]. An osteogenic response is more likely with high loads due to the triggering of higher mechanostat thresholds [34]. This could suggest that appropriate load bearing exercises such MST can promote increases in BMD, thereby reducing the risk for osteoporosis among PLWHIV.

Compared to findings of some previous studies [21, 35, 36], significant improvements in BMD were observed after a short strength exercise training programme of 12 weeks in the current study. This could be due to progressive loading and emphasis on intensity monitoring in the MST. In the current study, the training load was re-evaluated every week to ensure progressive loading throughout the intervention which maintains the mechanical stress that regulates bone remodeling through osteoblasts [13]. In addition, it has been reported that longer periods of more than 3 months of exercise interventions lead to high dropout rates [14, 37]. The adherence rate in the current study was 93% in the TG and 90% in the CG. The compliance rate was 100% with all participants completing all planned training sessions. Therefore, MST which promotes shorter exercise durations could be an alternative and attractive intervention for managing reduced BMD in PLWHIV.

Concurrent with previous findings [17, 18], significant improvements in muscle strength following MST were observed in the current study. Contrary to most conventional ways of strength training, MST emphasies on high loads and few repetitions with focus on high acceleration during the concentric phase which leads to high rate of force development as the muscles contaract [17]. However, while significant improvements in strength were observed in the TG only in the study by Mosti et al. (2013) [17], current findings reveal significant strength improvements in both the TG and CG as determined by increases in 1RM. Although both groups exhibited significant strength improvements, there were greater improvements among participants within the TG. Notable increases in strength in the CG in the present study could be attributed to occupational activities of most Malawian adults or other undetermined reasons. Data for the study was collected between June 2018 and March 2019 which falls within the farming period in Malawi. During this period most Malawians are involved in land preparation, planting and harvesting which demand a considerable physical capacity.

In line with other studies [25], significant reductions in heart rate were observed in the current study. A meta-analysis of randomised trials by O’Brien et al. (2008) reported a reduction in heart rate of – 13.2 bpm for participants in the TG. An almost similar reduction in heart rate (− 8.14 bpm) has been shown in the current study suggesting that MST has an effect on some cardiopulmonary parameters among PLWHIV.

To the best of our knowledge, this is the first randomised controlled trial that has evaluated the effects of MST exercises with focus on BMD as the main outcome in PLWHIV. A supervised, individualised and facility based MST exercise programme targeting BMD with clear descriptions of exercise type, frequency, intensity and duration was adopted. This pilot study provides preliminary data that allows potential for larger prospective studies on effective exercise strategies used to manage reduced BMD among PLWHIV. One limitation of the study was a relatively low sample size. However, based on previous studies [9, 17] the number of participants was adequate to assess the effectiveness of a MST programme on bone and strength parameters as revealed in the findings. Considering that gender may be a plausible confounder for bone responses to external loads, another limitation of the study was that analysis for BMD responses within gender were not done due to low sample size. Therefore, larger prospective trials with larger sample size investigating the effects of MST on BMD in PLWHIV are warranted.

## Conclusions

The study demonstrates that a 12 weeks’ exercise programme of MST improves lumbar BMD, heart rate and muscle strength in PLWHIV on ART exhibiting reduced BMD in Blantyre, Malawi. Data from this pilot study suggest that individualized and supervised MST performed for a shorter duration of 12 weeks seem to have the potential in treating reduced BMD among PLWHIV on ART. Since this was a pilot study, larger randomized controlled trials investigating the effects of 12 weeks MST on reduced BMD among PLWHIV with a larger sample size are merited.

## Data Availability

The data that support the findings of this study are available from the College of Medicine Ethics Committee (COMREC), but restrictions apply to the availability of these data, because the data contains potentially identifying information and so are not publicly available. Data are however available from the authors upon reasonable request and with permission of COMREC an IRB that approved the study.

## Abbreviations

1RM: One repetition Maximum
ART: Antiretroviral Therapy
BMD: Bone Mineral Density
CG: Control group
DEXA: Dual Energy X-ray Absorptiometry
HIV: Human Immunodeficiency Syndrome
MST: Maximal Strength Training
PLWHIV: People living with HIV
TG: Training group
